# Reactions of Subcutaneous Connective Tissue to Mineral Trioxide Aggregate, Biodentine®, and a Newly Developed BioACTIVE Base/Liner

**DOI:** 10.1155/2020/6570159

**Published:** 2020-05-18

**Authors:** Barış Karabulut, Nazmiye Dönmez, Ceren Canbey Göret, Cafer Ataş, Özlem Kuzu

**Affiliations:** ^1^Health Sciences University Faculty of Dentistry Department of Pedodontics, Istanbul, Turkey; ^2^Bezmialem Vakif University Faculty of Dentistry Department of Restorative Dentistry, Istanbul, Turkey; ^3^Health Sciences University Department of Surgical Pathology, Bagcilar Research and Education Hospital, Istanbul, Turkey

## Abstract

Aim. There is an increasing interest in the application of BioACTIVE materials to achieve hard tissue formation and maintain pulp vitality. Mineral trioxide aggregate (MTA) and Biodentine® are BioACTIVE materials used for pulp capping. Recently, dental researchers have produced BioACTIVE glass-incorporated light-curable pulp capping material. The study is aimed at evaluating the subcutaneous connective tissue reactions to MTA, Biodentine®, ACTIVA BioACTIVE Base/Liner. These materials were placed in polyethylene tubes and implanted into the dorsal connective tissue of Sprague Dawley rats. The presence of inflammation, predominant cell type, calcification, and thickness of fibrous connective tissue was recorded by histological examination 7, 30, and 60 days after the implantation procedure. Scores were defined as follows: 0 = none or few inflammatory cells, no reaction; 1 = <25 cells, mild reaction; 2 = 25 to 125 cells, moderate reaction; and 3 = ≥125 cells, severe reaction. Fibrous capsule thickness, necrosis, and formation of calcification were recorded. ANOVA and post hoc Dunnett's tests were used for statistically analyses (*p* < 0.05). Results. In terms of oedema, inflammation, fibrous capsule, and necrosis, no significant differences were found in any time period for any material. MTA and Biodentine® showed higher calcification than in the ACTIVA BioACTIVE on day 30, and the difference was statistically significant (*p* < 0.05). After 60 days, while calcification was not seen in the control group, it was observed in the test groups. There was a statistically significant difference between the control and the others. Conclusion. All materials were well tolerated by the tissues in the 60-day evaluation period. One notable finding is the presence of dystrophic calcification in the connective tissue adjacent to the newly developed BioACTIVE Base/Liner material. Therefore, this new BioACTIVE Base/Liner material may be safely recommended to clinicians as a pulp capping material.

## 1. Introduction

The main difficulty for the current approach in restorative dentistry is to provoke the remineralization of hypomineralized carious dentine, therefore protecting and preserving the vital pulp [1].

Vital pulp therapy is of great importance for preserving the tooth as it provides nutrition and defence and acts as a biosensor to detect pathogenic stimuli. Pulp capping is a treatment in which biocompatible agents are placed over the vital pulp to seal and protect it against bacterial penetration [2, 3]. The outcome of successful pulp capping is preservation of pulpal tissues and dentin bridge formation [4].

Calcium hydroxide has been the gold standard for pulp capping in recent decades [1, 4–6]; however, calcium hydroxide has some noticeable disadvantages, including inflammation and necrosis of the pulp surface after pulp capping, high solubility in oral fluids, degradation over time, insufficient adherence to dentinal walls, multiple tunnel defects inside the dentin bridge, and low mechanical resistance, which may cause the future failure of the treatment [1, 7, 8]. Experiences with calcium hydroxide as a lining material for restorations located close to the pulp have greatly influenced the initiative to develop BioACTIVE materials rather than just biocompatible ones [9, 10].

The biological compatibility of BioACTIVE materials is currently a topic of significant interest in the field of restorative dental medicine. The area of regenerative dentistry has been affected more by the use of BioACTIVE materials than by biocompatible materials [9, 10]. BioACTIVE materials can interact with the biological environment to evoke a specific biological response, such as the formation of a hydroxyapatite layer with a bond forming between the tissue and the material. The restorative materials currently available can simulate the tooth in appearance, form, and function but lack BioACTIVE properties. The development of dental restorative materials able to remineralize or repair demineralized dentin, following the bacterial attack, has been one of the areas of dental biomaterial research [11].

Currently, commonly used pulp capping materials include BioACTIVE hydraulic calcium silicate-based materials such as MTA [5] and Biodentine® [7]. Many studies have reported that MTA stimulates the production of reparative dentin formation without producing an inflammatory tissue response in the pulp [5]. MTA, composed of Portland cement and bismuth oxide, is the most famous of these cements and has been shown to induce reparative dentinogenesis in pulp capping [8, 12]. MTA produces a high alkaline pH and induces hard tissue formation. Previous investigations of the physical properties of MTA have demonstrated good marginal adaptation, good sealing ability [13, 14], and low or no solubility [15]. One of the definite features of MTA is that it is a hydraulic material which means it sets in the presence of moisture [16]. The indications for the use of MTA have expanded, and it has become a superior alternate for calcium hydroxide including direct and indirect pulp capping [1]. However, it presents different drawbacks consisting of handling difficulty, extended setting time [17], capacity to induce crown discoloration, poor mechanical properties, poor adhesion to dental tissue, and being expensive [6, 8]. Several new calcium silicate-based materials have been developed which are aimed at improving these disadvantages of MTA [1, 18–20].

Biodentine® (Septodont, Saint Maur des Fosses, France) is declared to be used as a dentin replacement material, in addition to having endodontic indications similar to those of MTA [16]. When compared to MTA, advances in Biodentine properties, such as setting time, mechanical qualities, and initial cohesiveness, led to a widened range of applications, including endodontic repair and vital pulp therapy [1]. It is resin-free and mainly composed of pure tricalcium silicate. The chemical composition differs from MTA by the addition of calcium carbonate to the powder. The liquid constituted a hydrated calcium chloride (as an accelerator to reduce setting time) and a water-reducing agent [21, 22]. It is considered an encouraging material due to its antimicrobial influence. Its antibacterial mechanism of action is achieved through its high pH and its ability to increase the osmotic pressure which can inhibit many bacteria and through inducing mineralization on bacterial surfaces [16]. Biodentine® has shown better compression and surface properties than other tricalcium silicate-based materials [23]. The usefulness of this material as a pulp capping agent has been demonstrated in humans and in rats [5, 8].

Recently, a BioACTIVE glass-incorporated light-curable pulp capping material, ACTIVA BioACTIVE Base/Liner (BA, Pulpdent, Watertown, MA, USA), was presented as a “light-cured resin-modified calcium silicate” (RMCS) combining uncompromised attributes of both composite and glass ionomer [1], but investigations of the biological activities in mammalian cells that result from its use are limited [24]. The material composed of diurethane and methacrylate-based monomers with a modified polyacrylic acid and polybutadiene-modified diurethane dimethacrylate (rubberized resin) and BioACTIVE glass as a filler [25]. It has greater release and recharge of calcium, phosphate, and fluoride than glass ionomers in a strong, resilient resin matrix that will not chip or crumble. It also stimulates apatite formation at the material-tooth interface. The base/liner adheres to dentin and does not require etching or bonding agents [26]. ACTIVA BioACTIVE does not contain any Bisphenol A, Bis-GMA, and BPA derivatives [27]. Sheng et al. and Daud et al. reported that BioACTIVE glass could promote mineral formation on dentin surfaces [28, 29]. BioACTIVE glass has been examined for pulp capping by virtue of its supposed dentinogenesis property. Gholami et al. reported that the ions released by the sol-gel nanoporous BAG particles did not inhibit the growth of human dental pulp stem cells but showed a high density of mineralized nodules [30].

Long et al. reported that sol-gel derived BioACTIVE glass, when used for direct pulp capping, stimulated the formation of a compact dentin bridge with inflammatory responses alike MTA, as shown in mechanically exposed pulps of rats [31]. In addition, they have reported that the extended setting time and undesired physical properties of MTA can be modified by the addition of BioACTIVE glass.

To this day, there is inadequate evidence and there have been too few clinical studies to support ACTIVA BioACTIVE's reliability in vital pulp therapies [1, 32–34], and no study was found about ACTIVA BioACTIVE Base/Liner's connective tissue reactions. Therefore, the aim of this study is to investigate the tissue reactions to ACTIVA BioACTIVE material, thereby determining whether it can be used as pulp capping material.

The null hypothesis is that there will be no difference in terms of biocompatibility between ACTIVA BioACTIVE Base/Liner material, ProRoot MTA®, and Biodentine**®**.

## 2. Material and Method

Ethical approval for this project was obtained on 7 April 2019 from the University of Health Sciences Hamidiye Animal Experiments Local Ethics Committee (reference number 10.04.2019/2019-04/07). Twenty-one male 4- to 6-month-old Sprague Dawley rats, weighing 250–280 g, were used in the study. The animals were housed in temperature-controlled cages and received water and food. Eighty-four polyethylene tubes with a 1.3 mm internal diameter, 1.6 mm external diameter, and 5 mm length were prepared. ACTIVA BioACTIVE Base/Liner, Biodentine**®**, and ProRoot MTA were prepared according to the manufacturers' recommendations and inserted into the tubes with a small condenser. The composition, batch numbers, and manufacturer of the materials are listed in Table 1. Twenty-one polyethylene tubes remained empty to be used as controls.

### 2.1. Subcutaneous Implant Test

The animals were shaved under xylazine (10 mg/kg) and ketamine (70 mg/kg) anesthesia. The shaved dorsal skin was disinfected with 5% iodine solution. Four incisions were performed on the animals' backs 2 cm from the spine and at least 2 cm apart. Using blunt-tipped scissors, the lateral tearing of the subcutaneous tissue provided 4 surgical cavities exposed in quadrants equidistant from the center of the animals' backs. Each animal received 4 tubes, and they were inserted into the surgical cavities parallel to the incision. The position in which each sealer was implanted was standardized. The incisions were closed using 3-0 silk thread.

The rats were further subdivided into 3 groups (7 days, 30 days, and 60 days) according to the time period of sacrifice. By the end of each period after surgery, 7 animals were sacrificed via an anesthetic overdose. Biopsies of skin and subcutaneous tissues (2 × 2 cm) containing the implants were obtained with 1 cm safety margins.

The subcutaneous tissues containing the tubes were excised and fixed in 10% neutral formalin for 48 h. After that, the specimens were trimmed parallel to the tube leaving at least 2 mm of tissue on each side and cut into two equal halves, and the tubes were removed. Then, the specimens were inserted in serial ascending concentration of ethyl alcohol for dehydration, followed by clearance in xylene, and embedded in paraffin at 58–62°C. Samples were cast parallel to the long axis of the tube to show the region of interest (tube opening); then, serial sections of 4 *μ*m thickness were prepared and stained with hematoxylin and eosin (H&E) stain to evaluate inflammatory reactions and new bone formation around the implanted materials.

Sections were examined under a light microscope (Nikon Ni-U Japan) at 40x, 100x, 200x, and 400x magnifications by an observer blind to all procedures involved.

Oedema was scored as follows: 0 = none, 1 = mild, 2 = moderate, and 3 = severe. Inflammatory reactions in the tissue in contact with the material on the open end of the tube were scored according to previous studies as follows: 0 = none or few inflammatory cells, no reaction; 1 = <25 cells, mild reaction; 2 = between 25 and 125 cells, moderate reaction; and 3 = 125 or more cells, severe reaction. Fibrous capsules were considered as follows: 1 = thin at <150 *μ*m and 2 = thick at >150 *μ*m. Calcification and necrosis were recorded as follows: 0 = absent or 1 = present.

### 2.2. Statistical analysis

IBM SPSS Statistics 20 software was used to perform statistical tests with the significance level set at 5%. The Shapiro–Wilk test was used to test normalcy; the values had normal distribution. Parametric tests were performed among the groups for pairwise comparisons (ANOVA and post hoc Dunnett's *t*-test). The results for all data were analysed at a significance level of *p* < 0.05.

## 3. Results

The distribution of oedema, inflammation, calcification, fibrous capsule, and necrosis on days 7, 30, and 60 is shown in Table 2.

### 3.1. Day 7

**Figure 1:**
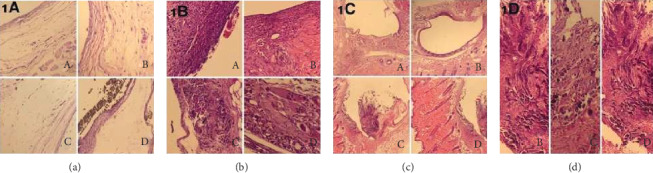
Photomicrograph of H&E staining showing the subcutaneous tissue of the control (A), Biodentine® (B), ProRoot MTA (C), and ACTIVA BioACTİVE Base/Liner (D) after 7 days exposure. (a) Oedema, (b) inflammation, (c) fibrous capsule, and (d) calcification. (a) Mild oedema especially around the fibrous capsule areas was observed in all groups (H&E ×200). (b) Loose connective tissue and mild inflammation limited to the tube end, and giant cells were present in all groups (H&E ×200 and ×400). (c) Thin fibrous capsule formation was observed in all groups (H&E ×40). (d) Dystrophic calcification was observed around and inside the fibrous capsule in the Biodentine® (B), ProRoot MTA (C), and ACTIVA BioACTİVE Base/Liner (D) groups. No calcification was seen in the control group (H&E x 200).

Mild oedema was observed in control group (5/7) specimens (Figure 1(a), A). All Biodentine® samples showed mild oedema (Figure 1(a), B). Mild oedema was also observed in the ProRoot MTA group (1/7) (Figure 1(a), C). All ACTIVA BioACTIVE Base/Liner samples showed mild oedema (Figure 1(a), D). Inflammatory cell infiltration was observed in both the control group (Figure 1(b), A) and the materials tested (Figure 1(b), B–D). Fibrous capsule formation was evident for most of the specimens in all groups (Figure 1(c), A–D). Calcification was not seen in control group samples. In the Biodentine® group (2/7) (Figure 1(d), B), ProRoot MTA group (2/7) (Figure 1(d), C), and ACTIVA BioACTIVE Base/Liner group (3/7) (Figure 1(d), D), dystrophic calcification was observed.

### 3.2. Day 30

**Figure 2:**
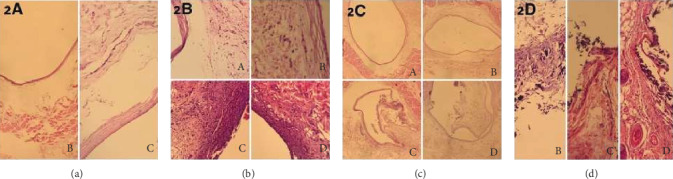
Photomicrograph of H&E staining showing the subcutaneous tissue of the control (A), Biodentine® (B), ProRoot MTA (C), and ACTIVA BioACTİVE Base/Liner (D) after 30 days exposure. (a) Oedema, (b) inflammation, (c) fibrous capsule, and (d) calcification. (a) Oedema was observed especially around the fibrous capsule in the ProRoot MTA (C) and ACTIVA BioACTİVE Base/Liner (D) groups (H&E ×100). (b) Loose connective tissue and mild inflammation were present in all groups around the fibrous capsule (H&E ×200). (c) Fibrous capsule formation was observed in all groups (H&E ×40). (d) Dystrophic calcification was observed around and inside the fibrous capsule in the Biodentine® (B), ProRoot MTA (C), and ACTIVA BioACTİVE Base/Liner (D) groups. No calcification was seen in the control group (H&E ×200).

Oedema results were similar for specimens of the ProRoot MTA and ACTIVA BioACTIVE Base/Liner groups (Grade 1 (3/7)) (Figure 2(a), C and D). The intensity of inflammation reduced in all groups. The Control group (2/7) (Figure 2(b), A), Biodentine® group (4/7) (Figure 2(b), B), ProRoot MTA group (3/7) (Figure 2(b), C), and ACTIVA BioACTIVE Base/Liner group (6/7) (Figure 2(b), D) specimens showed mild inflammatory cell infiltration. Fibrous capsule formation was observed in all groups (Figure 2(c), A–D). Calcification was not observed in the control group. But in the Biodentine® group (5/7) (Figure 2(d), B), ProRoot MTA group (7/7) (Figure 2(d), C), and ACTIVA BioACTIVE Base/Liner group (3/7) (Figure 2(d), D), dystrophic calcification was present.

### 3.3. Day 60

**Figure 3:**
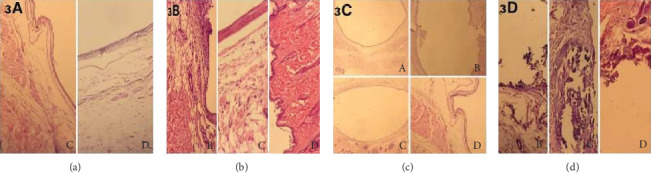
Photomicrograph of H&E staining showing the subcutaneous tissue of the control (A), Biodentine® (B), ProRoot MTA (C), and ACTIVA BioACTİVE Base/Liner (D) after 60 days exposure. (a) Oedema, (b) inflammation, (c) fibrous capsule, and (d) calcification. (a) Oedema was present especially around the fibrous capsule in the ProRoot MTA (C) and ACTIVA BioACTİVE Base/Liner (D) groups (H&E ×40). (b) Loose connective tissue and mild inflammation were observed around the fibrous capsule in the Biodentine® (B), ProRoot MTA (C), and ACTIVA BioACTİVE Base/Liner (D) groups. No inflammation was present in the control group (H&E ×100 and ×200). (c) Fibrous capsule formation was present in all groups (H&E ×40). (d) Dystrophic calcification was observed around and inside the fibrous capsule in Biodentine® (B), ProRoot MTA (C), and ACTIVA BioACTİVE Base/Liner (D) groups. No calcification was seen in the control group (H&E ×200).

Mild oedema was seen only in the ProRoot MTA (2/7) and ACTIVA BioACTIVE Base/Liner (3/7) groups (Figure 3(a), C and D). There was no inflammatory response in the control group. Specimens in the Biodentine® group (1/7) (Figure 3(b), B), ProRoot MTA group (1/7) (Figure 3(b), C), and ACTIVA BioACTIVE Base/Liner group (2/7) (Figure 3(b), D) were graded as 1. Thick fibrous capsule formation was evident for all groups (Figure 3(c), A–D). Calcification was not observed in the control group. But the Biodentine® group (5/7) (Figure 3(d), B), ProRoot MTA group (7/7) (Figure 3(d), C), and ACTIVA BioACTIVE Base/Liner group (5/7) (Figure 3(d), D) specimens showed dystrophic calcification.

### 3.4. Comparisons among Groups

There were no statistically significant differences at 7 days in all groups. The ProRoot MTA (1.00 ± 0.00) and Biodentine® (0.71 ± 0.48) groups showed higher calcification than the ACTIVA BioACTIVE Base/Liner (0.43 ± 0.53) group on day 30, and the difference was statistically significant (*p* < 0.05) (Table 3). After 60 days, while calcification was not seen in the control group, in the others, calcification was present. There were statistically significant differences between the controls and the others (*p* < 0.05) (Table 3).

## 4. Discussion

Pulp capping materials have been shown to play a vital role as restorative materials in the successful regeneration of the dentin-pulp complex. Pulp capping materials not only have pulp sealing effects but also have biological properties, such as biomineralization, which lead to dentin-pulp complex regeneration [35].

Direct and indirect pulp capping treatment is intended to preserve pulp vitality in selected cases. It has been shown that one of the most important properties of a pulp protecting material is its capacity to induce the formation of high-quality mineralized tissue [1, 36]. Our hypothesis in the current study was that there would be no differences in biocompatibility among ACTIVA BioACTIVE material, ProRoot MTA, and Biodentine®. According to these results, there were no significant differences among all tested materials, in terms of calcification, inflammatory response, and necrosis parameters (*p* > 0.05). Therefore, the null hypothesis is accepted.

In dentistry, evaluating the biocompatibility of new products has vital importance. Before marketing and using dental materials, it is mandatory to ensure that these materials have no side effects when in contact with tissues [37]. The International Organization for Standardization (ISO) standard 7405 determines test methods for dental material and preclinical evaluation of biocompatibility of medical devices used in dentistry [38]. This ISO standard governs the evaluation of the biological effects of dental materials [39]. All these materials come in contact with oral mucosa, dental pulp, and dental hard tissues [39]. Implantation in the subcutaneous tissue of rats is among the most convenient and relatively uncomplicated tests to determine the local effects of dental materials [40, 41]. It affords a comparative interpretation of data within one animal, with the lowest number of variables [42]. The inflammatory response is a characteristic phenomenon common to all fibrous connective tissue and varies little from tissue to tissue or from animal to animal among the higher species [42]. The *in vivo* subcutaneous implantation method provides sufficient information about inflammatory and immune responses induced by the test materials. The tissue responses to tested materials should be similar to the responses in the control for the material to be considered biocompatible and nontoxic [41]. The implantation of materials in polyethylene tubes has been widely accepted [43]. The tubes help fix the material at the site to maintain proper contact between the material and the tissues [33]. The implantation periods in this study were within the short- and long-term time intervals of the recommended standard practices for biological evaluation of dental materials [37]. In this study, we preferred to use the subcutaneous connective tissue method to evaluate calcification, inflammatory response, and necrosis parameters on ProRoot MTA, Biodentine®, and the newly developed ACTIVA BioACTIVE Base/Liner.

In the literature, for both MTA and Biodentine®, the calcium silicate-based cements used as pulp capping material are suggested for pulp capping treatment [44]. ACTIVA BioACTIVE Base/Liner is a new dental material recommended for pulp capping. The material has the advantage of stimulating mineral apatite crystal formation, and therefore, ACTIVA BioACTIVE Base/Liner material was included in this study to evaluate the reactions in subcutaneous tissue. While the use of MTA and Biodentine® is difficult to handle, ACTIVA BioACTIVE Base/Liner can be an alternative to these materials. Further, MTA and Biodentine® are expensive materials, suggesting an additional motivation to investigate whether ACTIVA BioACTIVE Base/Liner can be used as a pulp capping material.

The empty tubes used in the control group in this study caused few reactions in subcutaneous connective tissue in line with previously reported findings [40, 45].

Moretton et al. [46] examined the biocompatibility of MTA with subcutaneous and intraosseous implantation methods in rats. Tissue reactions were studied at 15, 30, and 60 days after implantation. Subcutaneous implants of MTA initially elicited severe reactions with coagulation necrosis and moderate dystrophic calcification, which subsided to mostly moderate, in time. Yaltirik et al. [45] examined histopathologically the biocompatibility of MTA and high-copper amalgam by implanting the test material into the subcutaneous connective tissue of rats for 7, 15, 30, 60, and 90 days. They reported that in MTA samples, although infiltration of inflammatory cells was lower at day 60, macrophages and giant cells were still phagocytosing the MTA particles in the connective tissue. The results of our study using MTA are compatible with those of Moretton et al. [46] and Yaltirik et al. [45].

Tran et al. [8] used Ca(OH)_2_, MTA, and Biodentine® in their study evaluating the biocompatibility of these materials. According to the data, they suggested that Biodentine® can be used in direct pulp capping. The current study found reactions to Biodentine® similar to those found by Tran et al. [8].

Similar moderate inflammatory tissue response was observed in the ACTIVA BioACTIVE Base/Liner, ProRoot MTA, and Biodentine® groups on day 7. At other time intervals in all groups, inflammatory cell numbers decreased compared to day 7. Mild inflammatory responses were recorded from all materials used in this study. These results for ProRoot MTA and Biodentine® are consistent with the findings of previous studies [5, 8]. Although the composition of ACTIVA BioACTIVE Base/Liner material is different from MTA and Biodentine®, similar tissue reactions were observed. This could be related to the silica particles common to all three materials.

ACTIVA BioACTIVE-Base/Liner was originated in 2014 declaring the strength, aesthetics, and physical properties and increased release and recharge of calcium, phosphate, and fluoride. Compared to both MTA and Biodentine, ACTIVA BioACTIVE represents a favourable setting time with no delay placing final restoration. But, the resin in pulp capping materials such as ACTIVA BioACTIVE BASE/LINER may lead to free monomers' release and consequently to pulpal toxicity [1].

According to Jun et al., ACTIVA exhibited the potential to stimulate biomineralization at the same level as MTA, Biodentine, and TheraCal LC on the basis of releasing the same amount of Ca and OH ions [24].

Comisi [33] reported that ACTIVA BioACTIVE Base/Liner material is used as an indirect pulp capping material to release calcium and phosphate ions to form apatite and help heal the tooth tissue. They also reported that the use of a BioACTIVE material that can reduce enzymatic initiation by the tooth and encourage biomineralization can provide benefits superior to traditional resin bonding procedures.

Koutroulis et al. compared the role of calcium ion release on biocompatibility and antimicrobial properties of several hydraulic cements, and the results showed that ACTIVA BioACTIVE Base/Liner presented characteristic microstructure of glass ionomer with negligible calcium release, acceptable biocompatibility, and moderate antibacterial activity [47].

Abou El Reash et al. [34] reported that MTA, Angelus HP, iRoot BP plus, and ACTIVA BioACTIVE Restorative material were used to compare the biocompatibility, in terms of inflammatory response, apoptotic activity, and healing ability of subcutaneous tissue implants in rats. They concluded that ACTIVA BioACTIVE exhibited excellent biocompatibility and healing ability for rat subcutaneous tissues, in comparison with calcium silicate-based cements. These results are compatible with the findings in our study.

Korkut et al. [48] compared the mechanical properties of four different resin-modified glass ionomers and found that ACTIVA BioACTIVE Restorative material met the requirements of minimum standards set by the ISO.

Omidi et al. [49] compared the microleakage of Class II (box only) cavity restorations with ACTIVA BioACTIVE Restorative, resin-modified glass ionomer, and composite in primary molars and observed that microleakage of ACTIVA BioACTIVE Restorative material was comparable to microleakage of composites in the absence or presence of etching and bonding. But, Alkhudhairy and Ahmad [50] reported a moderate level of microleakage in ACTIVA BioACTIVE Restorative glass in Class II (box only) cavities of maxillary premolars.

Sahoo et al. [51] compared the bond strengths of compomer, ormocer, nanofilled composite, and ACTIVA BioACTIVE conditioned in different solvents and found that nanofilled composite was significantly stronger than the ormocer and ACTIVA BioACTIVE. The compomer was found to be the weakest. They also stated that shear bond strength was significantly increased for ACTIVA BioACTIVE after conditioning in distilled water.

Although these studies have evaluated the mechanical properties of ACTIVA BioACTIVE material, it can be said that it is an acceptable material both mechanically and histologically in the dental application in the clinic.

Lopez-Garcia et al. [52] evaluated the biological effects of ACTIVA Kids BioACTIVE, and they found that ACTIVA displayed higher metabolic activity, cell migration, and better cell morphology indicating lower cytotoxicity than resin-modified glass ionomers.

## 5. Conclusion

Histological response to ACTIVA BioACTIVE Base/Liner was very similar to Biodentine® and ProRoot MTA. All materials were well tolerated by the tissues in the 60-day evaluation period. One notable result is the presence of dystrophic calcification in the connective tissue adjacent to the newly developed BioACTIVE Base/Liner material. Therefore, this new base/liner material may be a potential pulp capping material. However, to accurately assess ACTIVA BioACTIVE's reparative potential or influence on the vital pulp in pulp capping procedures, further *in vitro* and *in vivo* studies are necessary.

## Figures and Tables

**Table 1:** The composition, batch number and manufacturers of dental materials.

Product/batch/manufacturer	Composition
ACTIVA BioACTIVE Base/Liner 191009 Pulpdent, Watertown, MA, USA	Blend of diurethane and other methacrylates with modified polyacrylic acid (∼53.2%), silica (∼3.0%), and sodium fluoride (∼0.9%)
ProRoot MTA 0000192899 Dentsply, Tulsa, OK, USA	Tricalcium silicate (66.1%), dicalcium silicate (8.4%), tricalcium aluminate (2.0%), tetracalcium aluminoferrite, calcium sulphate bismuth oxide (14%), calcium oxide (8%), silicon oxide (0.5%), and aluminium oxide (1.0%)
Biodentine® B23071 Septodont, St. Maur des Fosses, FRANCE	Powder: tricalcium silicate (80.1%), dicalcium silicate, calcium carbonate (14.9%), iron oxide, and zirconium oxide (5%). Liquid: water, calcium chloride, and partially modified polycarboxylate

**Table 2:** Number of samples with oedema, inflammation, fibrous capsule, calcification, and necrosis scores on days 7, 30, and 60.

		Control				ACTIVA BioACTİVE Base/Liner				ProRoot MTA				Biodentine®			
Time		0	1	2	3	0	1	2	3	0	1	2	3	0	1	2	3
7 days	Calcification	7	—			4	3			5	2			5	2		
	Necrosis	7	—			7	—			7	—			7	—		
	Oedema	2	5	—	—	—	7	—	—	5	1	1	—	—	7	—	—
	Inflammation	0	5	2	—	—	1	5	1		1	5	1	—	4	3	—
	Fibrous capsule		7	—			3	4			3	4			1	6	
30 days	Calcification	7	—			4	3			—	7			2	5		
	Necrosis	7	—			7	—			7	—			7	—		
	Oedema	7	—	—	—	4	3			4	3			7			
	Inflammation	5	2	—	—	1	6			3	4			4	3		
	Fibrous capsule		—	7			—	7			—	7			—	7	
60 days	Calcification	7	—			2	5			—	7			2	5		
	Necrosis	7	—			7	—			7	—			7	—		
	Oedema	7	—	—	—	4	3	—	—	5	2	—	—	7	—	—	—
	Inflammation	7	—	—	—	5	2	—	—	6	1	—	—	6	1	—	—
	Fibrous capsule		—	7			—	7			—	7			—	7	

Oedema (0 = absent, 1 = mild, 2 = moderate, and 3 = severe), inflammatory response (0 = absent, 1 = mild, 2 = moderate, and 3 = severe), fibrous capsule (1 = thin at <150 *μ*m and 2 = thick at >150 *μ*m), calcification (0 = absent and 1 = present), and necrosis (0 = absent and 1 = present).

**Table 3:** Mean and standard deviation cell values of groups in all test periods.

Groups		Mean ± SD		
		7 days	30 days	60 days
Control	Calcification	0.00 ± 0.000	0.00 ± 0.000 A	0.00 ± 0.000 A
	Necrosis	0.00 ± 0.000	0.00 ± 0.000	0.00 ± 0.000
	Oedema	0.71 ± 0.488	0.00 ± 0.000	0.00 ± 0.000
	Inflammation	1.29 ± 0.488	0.29 ± 0.488	0.00 ± 0.000
	Fibrous capsule	1.00 ± 0.000	1.00 ± 0.000	1.00 ± 0.000
ACTIVA BioACTIVE Base/Liner	Calcification	0.43 ± 0.535	0.43 ± 0.535 A	0.71 ± 0.488 B
	Necrosis	0.00 ± 0.000	0.00 ± 0.000	0.00 ± 0.000
	Oedema	1.00 ± 0.000	0.43 ± 0.535	0.43 ± 0.535
	Inflammation	2.00 ± 0.577	0.86 ± 0.378	0.29 ± 0.488
	Fibrous capsule	0.57 ± 0.535	1.00 ± 0.000	1.00 ± 0.000
ProRoot MTA	Calcification	0.29 ± 0.488	1.00 ± 0.000 B	1.00 ± 0.000 B
	Necrosis	0.00 ± 0.000	0.00 ± 0.000	0.00 ± 0.000
	Oedema	1.00 ± 0.577	0.43 ± 0.535	0.29 ± 0.488
	Inflammation	2.00 ± 0.577	0.57 ± 0.535	0.14 ± 0.378
	Fibrous capsule	0.57 ± 0.535	1.00 ± 0.000	1.00 ± 0.000
Biodentine®	Calcification	0.29 ± 0.488	0.71 ± 0.488 B	0.71 ± 0.488 B
	Necrosis	0.00 ± 0.000	0.00 ± 0.000	0.00 ± 0.000
	Oedema	1.00 ± 0.000	0.00 ± 0.000	0.00 ± 0.000
	Inflammation	1.43 ± 0.535	0.43 ± 0.535	0.14 ± 0.378
	Fibrous capsule	0.86 ± 0.378	1.00 ± 0.000	0.86 ± 0.378

There is no difference between the same letters in the same column.

## Data Availability

The data used to support the findings of this study are included within the article.
